# Key Challenges for Respiratory Virus Surveillance while Transitioning out of Acute Phase of COVID-19 Pandemic

**DOI:** 10.3201/eid3002.230768

**Published:** 2024-02

**Authors:** Oliver Eales, Michael J. Plank, Benjamin J. Cowling, Benjamin P. Howden, Adam J. Kucharski, Sheena G. Sullivan, Katelijn Vandemaele, Cecile Viboud, Steven Riley, James M. McCaw, Freya M. Shearer

**Affiliations:** The University of Melbourne, Melbourne, Victoria, Australia (O. Eales, B.P. Howden, S.G. Sullivan, J.M. McCaw, F.M. Shearer);; Imperial College London, London, UK (O. Eales, S. Riley);; University of Canterbury, Christchurch, New Zealand (M.J. Plank);; World Health Organization Collaborating Centre for Infectious Disease Epidemiology and Control, The University of Hong Kong, Hong Kong, China (B.J. Cowling);; Centre for Epidemic Preparedness and Response, London School of Hygiene and Tropical Medicine, London (A.J. Kucharski);; Global Influenza Programme, World Health Organization, Geneva, Switzerland (K. Vandemaele);; Fogarty International Center, National Institutes of Health, Bethesda, Maryland, USA (C. Viboud)

**Keywords:** COVID-19, surveillance, SARS-CoV-2, pandemic, coronavirus disease, severe acute respiratory syndrome coronavirus 2, viruses, respiratory infections, zoonoses, vaccine-preventable diseases

## Abstract

To support the ongoing management of viral respiratory diseases while transitioning out of the acute phase of the COVID-19 pandemic, many countries are moving toward an integrated model of surveillance for SARS-CoV-2, influenza virus, and other respiratory pathogens. Although many surveillance approaches catalyzed by the COVID-19 pandemic provide novel epidemiologic insight, continuing them as implemented during the pandemic is unlikely to be feasible for nonemergency surveillance, and many have already been scaled back. Furthermore, given anticipated cocirculation of SARS-CoV-2 and influenza virus, surveillance activities in place before the pandemic require review and adjustment to ensure their ongoing value for public health. In this report, we highlight key challenges for the development of integrated models of surveillance. We discuss the relative strengths and limitations of different surveillance practices and studies as well as their contribution to epidemiologic assessment, forecasting, and public health decision-making.

Surveillance plays a critical role in the management of epidemic diseases. This role has most recently been demonstrated by the COVID-19 pandemic, during which existing approaches to respiratory pathogen surveillance, such as community testing, were rapidly scaled up and many enhanced or new surveillance activities, such as infection prevalence surveys, were put into place (*1*). The data generated by COVID-19 surveillance systems have provided situational awareness and informed myriad policy questions (*2*,*3*). The unprecedented circumstances of the pandemic revealed both surveillance system strengths, such as where required data were available in a timely manner, and shortcomings, including where data were delayed, unavailable, or uninformative.

Many components of COVID-19 surveillance systems that were established to support pandemic response provided insight into epidemic dynamics and intervention ramifications beyond those provided by surveillance systems in place before the pandemic. For example, in the United Kingdom, national infection prevalence surveys provided near–real-time insight into the infection (as opposed to case) dynamics of SARS-CoV-2 (*4*,*5*), including infection rates over time by age group (*6*). Household studies and the systematic collection of contact tracing data, particularly in Europe (*7*) and Asia (*8*), enabled estimation of key biological quantities affecting transmission (e.g., the generation interval). Systematic collection of behavioral data from population surveys—with prominent examples in Australia (*9*), Hong Kong (*10*), and the United Kingdom (*11*)—provided information on the influence of public health measures, including the functioning of various surveillance components. International and country-level platforms for genomic data integration and reporting enabled early characterization of variants of concern and offered insight into the global patterns of variant spread (*12*–*15*).

As countries transition out of the pandemic, many have discontinued or scaled back COVID-19 surveillance activities and are moving toward integrated surveillance for COVID-19, influenza, and other viral respiratory illnesses of epidemic or pandemic potential (*16*). Globally, COVID-19 continues to place a substantial burden on population health and health systems (*17*). Seasonal patterns are not yet possible to predict, but future epidemic waves of SARS-CoV-2 are anticipated (*18*), and we continue to face the threat of novel variants. As social disruption resulting from COVID-19 decreases, seasonal circulation patterns of other respiratory viruses have resumed (*19*). The long-term effect of cocirculation and behavioral- and immune-mediated interactions between SARS-CoV-2 and other respiratory pathogens is unknown but is expected to place additional pressure on healthcare systems. Effective surveillance will help healthcare officials to rapidly gauge the status of concurrent epidemics during interpandemic periods and make appropriate preparations for the healthcare systems that might be taxed. To assess, anticipate, and respond to this overall viral respiratory disease burden, it is important to monitor the individual dynamics of each pathogen. Effective surveillance will also enhance levels of preparedness for responding to the next (inevitable) global respiratory virus emergency, including establishing criteria for activating (and deactivating) enhanced surveillance activities (e.g., special studies) according to surveillance objectives. Surveillance objectives will vary by locality and epidemiologic context (as explored by the World Health Organization’s Mosaic framework), but a collaborative surveillance approach will support national, regional, and global preparedness (*20*,*21*).

To inform the design of a sustainable, integrated model of viral respiratory pathogen surveillance for interpandemic management (Figure), now is a critical time to review both surveillance practices in place before the pandemic and novel approaches adopted for pandemic response. Resuming pathogen-specific surveillance approaches, such as those for monitoring influenza, would represent a missed opportunity to build on learnings from emergency response efforts. We describe key challenges in designing integrated viral respiratory pathogen surveillance as the world transitions out of the COVID-19 pandemic. We focus on challenges that apply to the monitoring of any individual viral respiratory pathogen as well as those that arise due to the cocirculation and integrated monitoring of multiple viruses. Furthermore, we explore how additional surveillance methods and studies can enhance knowledge during interpandemic periods and support the ability of healthcare officials to anticipate and appropriately mitigate viral threats.

**Figure:**
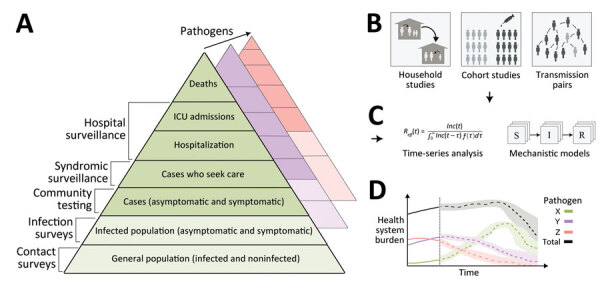
An integrated surveillance system for multiple circulating respiratory pathogens. A) Surveillance systems, which collect data continuously, monitor changes in infection outcomes (or behavior) at different levels of severity on the severity pyramid. In general, the proportions in each level can vary over time (e.g., because of emerging new variants), so surveillance should be undertaken at multiple levels. Collecting additional behavioral data can help disentangle changes in the data streams resulting from behavior specifically (e.g., changes in healthcare-seeking behavior) or indirectly (e.g., changes in transmission rates due to changes in contact rates). In addition, collecting data on interventions (e.g., vaccine uptake, therapeutic use) at all levels of the severity pyramid can help to enable estimates of intervention effectiveness. In general, multiple pathogens will circulate concurrently, and it may be necessary for surveillance systems to be extended to distinguish between the different pathogens. Integrating genomic sequencing of samples collected at all levels can additionally allow differences in dynamics between variants to be identified. B) Enhanced surveillance activities conducted periodically can augment continuous surveillance. Enhanced surveillance activities may inform on key epidemiologic quantities, such as the generation interval and vaccine effectiveness, which are important for predicting future transmission dynamics. C) Data collected from different sources of clinical, epidemiologic, and genomic surveillance can be synthesized through data analysis pipelines and used to generate epidemic forecasts and scenario projections (long- and short-term). D) Forecasts and projections can be used to support public health decision-making and planning. For example, they can be used to predict the timing and size of concurrent epidemics of influenza, SARS-CoV-2, and RSV and anticipate the resulting combined healthcare burden. ICU, intensive care unit; S→I→R, susceptible-intermediate-resistant model.

## Measuring the Clinical Burden of Multiple Circulating Respiratory Pathogens

### How Can Sentinel Syndromic Surveillance Handle Multiple Pathogens with Overlapping Symptom Profiles?

In many parts of the world, the public health response to COVID-19—including stay-at-home orders (*22*,*23*) and a requirement to isolate if testing positive—strongly suppressed viral respiratory transmission (*9*). Accordingly, cocirculation of such other respiratory pathogens as influenza and respiratory syncytial virus was limited (Burstein R et al, unpub. data, https://doi.org/10.1101/2022.02.04.22270474) (*24*). In the absence of other circulating pathogens, interpretation of syndromic surveillance data was simplified given the high likelihood of SARS-CoV-2 as the causative virus in persons reporting symptoms. Conversely, in the context of cocirculating pathogens, interpretation of data from syndromic surveillance is more complicated; the overall clinical burden will often be the result of multiple concurrent epidemics. As a case in point, the 2022 autumn season in the United States was characterized by large, overlapping epidemics of influenza virus, respiratory syncytial virus, and SARS-CoV-2 infections, exerting unusual pressure on intensive care unit capacity, a phenomenon coined as the tripledemic (*25*).

Before the COVID-19 pandemic, surveillance of seasonal influenza primarily relied on sentinel syndromic surveillance, where a fraction of healthcare sites routinely reported the daily number of persons seeking treatment for influenza-like illness (ILI) (*26*,*27*). ILI-defining symptoms are not influenza specific, and the temporal signal can be biased by other respiratory pathogens with similar symptom profiles (*28*,*29*). For this reason, a subset of ILI cases undergo testing to estimate the proportion for which influenza was the causative virus (ILI+). The overlapping symptom profiles of COVID-19 and influenza will likely result in an increase in the number of persons categorized as symptomatic for ILI (i.e., decreasing the specificity of ILI-defining symptoms for identifying influenza infection), and previously suitable sampling strategies for testing may no longer yield a sufficient sample size for reliable evaluation of each virus’s contribution to overall burden.

Expanding influenza sentinel syndromic surveillance to incorporate COVID-19 surveillance, as proposed by the World Health Organization (*16*), would avoid the need for 2 partially redundant surveillance systems. Just as for seasonal influenza surveillance, ILI definitions commonly used for reporting would not (nor would be required to) capture all symptomatic COVID-19 cases (*30*). Broadening the symptom criteria (and increasing the total sample) for routine reporting would capture a greater fraction of symptomatic infections, irrespective of the underlying virus. However, because this change could reduce the specificity of the symptom criteria, a more conscientious approach might be to investigate how the symptom criteria could be optimized to provide the most epidemiologic insight, while still maintaining cost-effective sampling rates (low specificity would require greater sampling rates). Because any syndromic signal detected would include infections caused by influenza virus, SARS-CoV-2, and other pathogens, a random subset of persons meeting the symptom criterion could undergo testing to identify the potential causative virus(es) (*31*). This process would enable the case time-series for each circulating pathogen to be resolved, improving prediction of individual and overall disease burdens (*29*) (Figure, panel D).

### How Can Trends in Community Levels of Infection Be Monitored?

Sentinel syndromic surveillance of ILI and ILI+ are typically assumed to be proportional to community levels of infection (*32*), despite those indicators only capturing infected persons who report to a healthcare provider. As was already established before the COVID-19 pandemic, the subset of the infected population seeking healthcare will be a biased subsample of the total infected population, and there is evidence of substantial interseason and intraseason variation in the fraction of all infections detected for various reasons (*33*). Hence, developing systems for monitoring the underlying levels of infection in the general population is important in providing a less biased, more stable denominator for assessments of clinical severity, estimates of intervention effectiveness, and forecasts and projections.

Estimating the true levels of infection is difficult and would require testing a representative sample of the general population regularly. However, if quantities (e.g., case notifications, hospitalizations, and deaths) that reliably correlate with the population level of infection can be monitored (and their biases understood), the underlying dynamics of transmission can be estimated. This process is not straightforward: first and foremost, case time-series data will not necessarily correlate reliably because of many factors, including variation in healthcare- and test-seeking behavior (*34*). During the COVID-19 pandemic, many surveillance data streams, including those from wastewater surveillance, symptom-based participatory studies, and mass testing, were used as correlates of SARS-CoV-2 infection, but not all of them will be sustainable in the future (Appendix, section A), and many have been already scaled back. Furthermore, whereas measuring correlates of infection rates is useful for estimating how they vary over time (i.e., relative changes), those measurements do not provide estimates of the true level of infection in the population over time.

An alternative approach to estimate the level of infection is to measure it directly. Random population testing is a key method that has been used in studies to measure infection prevalence of SARS-CoV-2 (*5*,*35*) and has been proposed for the ongoing surveillance of SARS-CoV-2 and other respiratory pathogens (*36*). Those studies enable robust estimation of the time-series of past infections and, in turn, the proportion of the population who are no longer susceptible to infection becaise recent exposure, improving epidemic forecasts and scenario projections (*37*). Such studies can be extended (or integrated with other data sources) to gain greater insight into key epidemiologic categories, including real-world vaccine effectiveness (*38*,*39*), the transmission advantage of new variants (*40*,*41*), symptomatology (*30*), and postviral sequelae (*42*). Potential challenges associated with such studies include low participation rates and high financial costs (Appendix, section B).

## Assessing Changes in Key Biologic Quantities

### How Can Key Epidemiologic Quantities Be Inferred?

Surveillance approaches that collect information on pairs of infectors and infectees in successive chains of transmission are required for directly estimating (and monitoring) several key biological measurements that drive epidemic dynamics, including the incubation period, generation interval, and relative contagiousness of asymptomatic infections (*43*). For example, the generation interval can most directly be estimated by linking dates of infection onset for infector–infectee pairs, data not collected through traditional case-based surveillance. Contact tracing activities and outbreak investigations do typically collect relevant data (*8*); however, those activities are not routine for patients with viral respiratory infections (outside of a pandemic), leaving a potential knowledge gap during interpandemic periods. Knowledge of the generation interval is required for accurate estimation of the effective reproduction number (the expected number of secondary infections caused by a primary infection) (*44*), which is critical in evaluating the likely effect of alternative control strategies. If studies were specifically designed to collect data on infector–infectee pairs, or embedded within other studies (e.g., infection prevalence surveys), then the availability of data would not depend on the presence of contact tracing or outbreak investigations (although the value of such studies would depend on the epidemic situation). Data quality would likely be enhanced given the more precise recording of time intervals associated with likely infection and symptom onset.

The generation interval of SARS-CoV-2 changed as the pathogen evolved (*45*,*46*) and as viewed under different policy (*8*) and transmission (*45*) settings. If surveillance were augmented by genomic data (Appendix, section C) from infector–infectee pairs during periods of variant emergence, changes in the generation interval between variants could be measured in real time and used to assess potential mechanisms of transmission advantage. This process also would enable estimation of the effective reproduction numbers of circulating variants during periods of variant replacement (*47*), improve real-time forecasts of variant outbreaks, and inform intervention planning. Genomic data may also improve accuracy of the inferred transmission links, for example, by excluding coincidental infections in close contacts that were actually acquired from independent sources.

### How Can a Virus’ Clinical Severity Be Quantified?

To predict the future number of severe outcomes (e.g., death, hospitalization), the relationship between infection and the probability of a severe outcome must be well characterized, including how it varies with age, vaccination status, comorbidities, and other epidemiologic covariates. Such predictions would require accurate estimates of infection levels (to compare with levels of severe outcomes) (*48*) or infection outcomes of an unbiased sample of infected persons.

The infection fatality ratio (IFR) is a measure of the proportion of infections that result in death; the infection hospitalization ratio (IHR) is the measure of the proportion of infections that result in hospitalization. Over the course of the COVID-19 pandemic, the IFR and IHR for SARS-CoV-2 changed as a result of mass vaccination and the emergence of new variants (*48*). For seasonal influenza, in countries with suitable data systems and resources, the case-fatality ratio and case hospitalization ratio are routinely computed and typically vary from season to season (*49*). It is, however, difficult to distinguish the extent to which changes in those case-based ratios for seasonal influenza result from changes in case ascertainment or the intrinsic biology of the circulating strain and effectiveness of the vaccine. The IFR and IHR for seasonal influenza (which are independent of case ascertainment) are seldom known because the underlying levels of infection in the community are rarely measured and hospitalizations and deaths are not always attributed to a specific pathogen, undercounting the true burden. The ability to measure any changes in the IFR and IHR of a pathogen is critical for their incorporation into situational assessments and scenario planning (*37*). Severe outcomes are recorded by public health systems in many countries, but their definitions may vary between (and within) countries or over time and often are not universal but rather sentinel-based or only in place for a subset of hospitals. Finally, mortality surveillance is often only available in near–real time for all-cause mortality, rather than for disease-specific mortality.

Linking the data collected on severe outcomes (and key covariates such as comorbidities) to surveillance systems that identify the levels of infection in the general population could more easily produce estimates of the IFR and IHR. If the definitions used for severe outcomes were standardized through time, then changes in severity between and within epidemic seasons could be more easily detected.

## Quantifying Changes in Epidemiologic Dynamics

### How Can Infection History Be Quantified?

Population immunity (i.e., the combined effect of individual-level immune responses) against SARS-CoV-2 infection (and reinfection) has increased in complexity through the course of the pandemic. At the beginning of the pandemic, there was likely minimal population immunity (i.e., almost all persons were susceptible to infection). Now, considering the availability of multiple vaccination courses, the emergence and spread of multiple variants, and waning of both vaccine- and infection-induced immunity, the individual- and population-level characteristics of immunity have become highly complex (*50*). Repeated epidemics of Omicron subvariants indicate that immunity against reinfection may not be long lasting, but reduced severity of infections over time is consistent with longer-lasting immunity against severe disease (*51*). In principle, all possible combinations of exposures and vaccinations, and their timing, must be considered to estimate individual-level and population immunity. As future variants and updated vaccines arrive, the problem will undergo a combinatorial explosion. This challenge is a known problem for influenza, and some modeling approaches are designed to manage this complexity (*52*).

Critical improvements for both influenza and COVID-19 could be made to forward projections of clinical burden and the design of future vaccination campaigns if the immune landscape could be more accurately quantified. Serologic data, which are used to measure the presence of specific antibodies in a person’s blood, can be used to quantify immunity when the antibodies correlate sufficiently with protection against infection (*53*). Serologic studies of influenza virus have greatly improved the understanding of long-term dynamics of influenza antibody-mediated immunity (*54*), but such serologic data have not been used for routine surveillance. Collecting serologic data was commonplace during the COVID-19 pandemic (*55*,*56*). Studies found that serum antibody titers correlated with protection against symptomatic infection during the first 2 years of the pandemic (*57*,*58*) and that neutralizing antibodies offered the strongest correlation (*59*,*60*). Most of the global population will now have SARS-CoV-2 antibodies in their serum (after infection, vaccination, or both), but future infections are likely to be attributable to variants able to evade those existing antibodies. In addition, the long-term dynamics of immunity against SARS-CoV-2 are not yet understood; correlate-of-protection studies (e.g., longitudinal cohort studies) should be established to identify appropriate immunologic tests that can quantify population- or individual-level immunity against infection with future SARS-CoV-2 variants. If suitable correlates-of-protection could be identified, then serologic surveillance systems could be established to quantify population immunity against newly emerging variants before waves of infection occur. If those immunologic observations could be combined with predictions of strain evolution (*61*,*62*), the future burden of respiratory viruses could be anticipated.

### Can the Frequency and Timing of Recurrent Epidemics Be Projected?

Long-term projections of the potential magnitude and timing of future epidemic seasons can assist health systems in better preparing for the resulting clinical burden. This work is distinct from (and complementary to) the statistical near-term forecasting of peak timing and size routinely conducted throughout an epidemic season (*63*).

Influenza transmission is highly seasonal; in temperate regions there are yearly winter epidemics (*64*), whereas in tropical regions there is a high background level of influenza infection and additional epidemics occur with less regular timing (*65*). The long-term dynamics of SARS-CoV-2 remain uncertain, but recurrent epidemics are likely to occur (*66*,*67*). In situations with substantial circulation of SARS-CoV-2, influenza virus, and other respiratory pathogens, it is not yet known how concurrent epidemics will interact. Virus–virus interference at the individual host level is a well-established phenomenon (*68*–*70*), but far less is understood about the epidemiologic consequences of that interference (*64*). Will concurrent epidemics cause increased pressure on health services? Or could epidemics interfere with each other, resulting in misalignment or less predictable behavior? Multiple recurrent epidemics involving SARS-CoV-2 and other established viruses will need to be observed to answer those questions. In the meantime, surveillance planning should consider such longer-term surveillance objectives. It is important that any data collected from surveillance systems are consistent between years, so that dynamics between years can be reliably compared and such questions over interepidemic timescales can be answered. In addition, data should be collected on potential drivers of changes in future epidemic dynamics (e.g., human contact networks, heterogeneities in age-specific transmission) so their effects can be quantified and incorporated into epidemic analysis (Appendix, section D). If serologic data were routinely collected, it might also be possible to regularly estimate population immunity, informing estimates of epidemic timing and size. Studies would first be required to identify when and what serologic data should be collected and by how much that information could improve estimates of epidemic timing.

Although respiratory viruses exhibit global transmission patterns, surveillance systems are largely designed and implemented at the local scale (e.g., country, state) to support local public health decision-making. Monitoring the transmission dynamics in other regions often can help predict future local dynamics (Appendix, section E). Finally, consideration should be given into how local surveillance data and data analyses could be shared between locales (in a timely manner) to improve global and local public health strategies.

## Conclusions

As the world transitions out of the acute phase of the COVID-19 pandemic, international and national surveillance systems are developing integrated systems of surveillance for SARS-CoV-2, influenza virus, and other viral respiratory pathogens. This integration requires a reevaluation of the public health objectives of surveillance and consideration of how both prepandemic practices and new approaches adopted during the COVID-19 pandemic can best support those objectives. We have highlighted how different surveillance practices, previously applied to influenza or COVID-19, contribute to specific areas of epidemiologic analysis and insight. We have identified challenges associated with respiratory virus surveillance, many of which relate to the monitoring of individual viral respiratory pathogens, whereas others are more specific to the development of integrated systems of surveillance. 

Open questions remain on the design of integrated models of surveillance, including a need to further optimize individual components and identify synergies and redundancies across them. Furthermore, the cost-effectiveness and feasibility of surveillance approaches in interpandemic and pandemic situations requires further investigation, as does the transferability of information across countries and regions. Our insights can assist surveillance planners as they assess the public health value and costs of various surveillance practices against the objectives of integrated surveillance for SARS-CoV-2, influenza virus, and other viral respiratory pathogens, supporting interpandemic management and preparedness for the next pandemic.
